# Calcineurin/NFAT signaling in the temporal integration of Ca²⁺ stress in neurodegeneration

**DOI:** 10.1038/s41420-026-03251-3

**Published:** 2026-07-11

**Authors:** Bruna Da Silva Oliveira, Metello Innocenti, Francesca Granucci

**Affiliations:** Department of Biotechnology and Biosciences, University of Milano-Bicocca, Milan, Italy

**Keywords:** Neuroscience, Immunology

## Abstract

The calcineurin (CaN)/nuclear factor of activated T cells (NFAT) signalling axis is a Ca²⁺-responsive pathway that translates intracellular Ca²⁺ signals into long-term transcriptional programmes. Chronic disruption of intracellular Ca²⁺ homoeostasis is a convergent feature of neurodegenerative disorders, particularly Alzheimer’s disease (AD) and Parkinson’s disease (PD). In these conditions, sustained or repetitive Ca²⁺ elevations promote prolonged activation of the CaN/NFAT pathway, thereby linking Ca²⁺ dysregulation to persistent cellular responses. In this review, we summarise the molecular organisation and regulation of the Ca²⁺/CaN/NFAT pathway and discuss its physiological roles in neurons and glial cells, including synaptic plasticity, neurodevelopment, neurogenesis, and neuroinflammatory responses. We critically examine experimental evidence linking CaN/NFAT signalling to AD and PD, distinguishing direct mechanistic roles from associative and model-dependent findings. Across disease contexts, the CaN/NFAT axis appears to function as a molecular node at which diverse insults, including amyloid-β and tau aggregates, α-synuclein toxicity, mitochondrial dysfunction, and chronic inflammatory cues, converge under conditions of sustained Ca²⁺ dysregulation. We propose that the pathological relevance of CaN/NFAT lies less in pathway activation per se than in its capacity to convert chronic Ca²⁺-dependent stress signals into persistent transcriptional states affecting synaptic integrity, inflammatory tone, and cellular resilience. We conclude by discussing current therapeutic strategies targeting this pathway, their limitations, and the need for temporally and cell-type-specific modulation.

## Facts

- Chronic Ca²⁺ dyshomeostasis is a recurrent feature of neurodegenerative disorders and provides a common condition for sustained activation of the CaN/NFAT signalling axis.
- CaN/NFAT signalling is unlikely to act as a primary initiator of AD or PD; rather, it functions as a stress-responsive node that links prolonged Ca²⁺ disturbances to long-term transcriptional changes.
- In neurons, sustained CaN/NFAT activation is associated with altered synaptic maintenance, dendritic remodelling, and reduced cellular resilience.
- In astrocytes and microglia, NFAT signalling shapes the duration and intensity of neuroinflammatory responses, acting in concert with pathways such as NF-κB.
- Therapeutic targeting of this pathway requires temporal and cell-type-specific precision, because broad CaN inhibition may disrupt essential physiological functions in the CNS.

## Open questions

- When does CaN /NFAT activation shift from an adaptive response to a maladaptive transcriptional programme in neurodegeneration?
- Which neuronal and glial populations are most vulnerable to maladaptive NFAT activation in AD and PD?
- How do different sources of Ca²⁺ dysregulation, including receptor-mediated entry, mitochondrial dysfunction, membrane disruption, and calpain-dependent calcineurin activation, shape NFAT-dependent transcription?
- Can selective or temporally restricted modulation of CaN/NFAT signalling prevent pathological persistence while preserving physiological Ca²⁺-dependent functions?
- Which biomarkers could identify patients or disease stages most likely to benefit from CaN/NFAT pathway modulation?

## Introduction

Neurodegenerative disorders, particularly Alzheimer’s disease (AD) and Parkinson’s disease (PD), represent a growing medical and societal challenge worldwide [1] for which effective disease-modifying therapies remain unavailable [2–6]. Substantial progress in elucidating disease-associated mechanisms such as protein misfolding, amyloid and tau pathology, oxidative stress, synaptic dysfunction and neuronal loss has been made in recent years.

Among them, chronic disruption of intracellular calcium (Ca²⁺) homoeostasis has emerged as a recurring feature of neurodegeneration [7, 8]. Ca²⁺ is a ubiquitous second messenger that links extracellular stimuli to intracellular signalling pathways and plays a central role in neuronal excitability, synaptic plasticity and gene expression, as well as in glial cell function [9]. Alterations in Ca²⁺ dynamics have been reported in both AD and PD, where sustained elevations of intracellular Ca²⁺ are thought to exacerbate synaptic failure, mitochondrial dysfunction and neuroinflammatory responses [10, 11].

A key molecular effector translating Ca²⁺ signals into long-lasting cellular responses is the calcineurin (CaN)/nuclear factor of activated T cells (NFAT) signalling pathway. CaN is a Ca²⁺/calmodulin-dependent phosphatase that responds to the amplitude and duration of intracellular Ca²⁺ signals and regulates the nuclear translocation and transcriptional activity of NFAT family members. In the central nervous system (CNS), CaN/NFAT signalling has been implicated in the regulation of neuronal survival, synaptic plasticity and glial inflammatory programmes [12, 13].

While the CaN/NFAT signalling axis is essential for physiological brain function, its sensitivity to Ca²⁺ dynamics also makes it vulnerable to pathological activation [14]. Indeed, sustained alterations of intracellular Ca²⁺ concentration can lead to prolonged CaN activation and persistent NFAT-dependent transcription, potentially linking synaptic dysfunction to maladaptive neuroinflammatory amplification [15]. Therefore, CaN and NFAT are unlikely to be a primary cause of neurodegeneration, but they more realistically represent a central signalling node through which diverse pathological insults, such as amyloid-β and tau-associated stress in AD, α-synuclein toxicity and mitochondrial Ca²⁺ burden in PD, converge to trigger shared transcriptional and cellular responses [13].

In this review, we summarize the molecular organisation and regulation of the Ca²⁺/CaN/NFAT pathway and discuss its physiological roles in neurons and glial cells. We also emphasise recent advances that refine the causal chain between pathological Ca²⁺ dyshomeostasis and CaN/NFAT-dependent transcription. These include membrane-level mechanisms of Aβ-induced Ca²⁺ entry, and proteolytic mechanisms that may convert transient CaN activation into persistent phosphatase activity. We then evaluate experimental evidence implicating CaN/NFAT signalling in the pathogenesis of AD and PD, highlighting where data support direct mechanistic links versus associative or model-dependent relationships. Finally, we discuss therapeutic strategies aimed at modulating this pathway, from classical CaN inhibitor, including preclinical low dose therapeutic treatments, to NFAT-selective approaches and Ca²⁺-modulating interventions, with particular attention to their translational potential and limitations in the central nervous system.

## Calcineurin: structure, regulation and functional role in the CNS

**Fig. 1:**
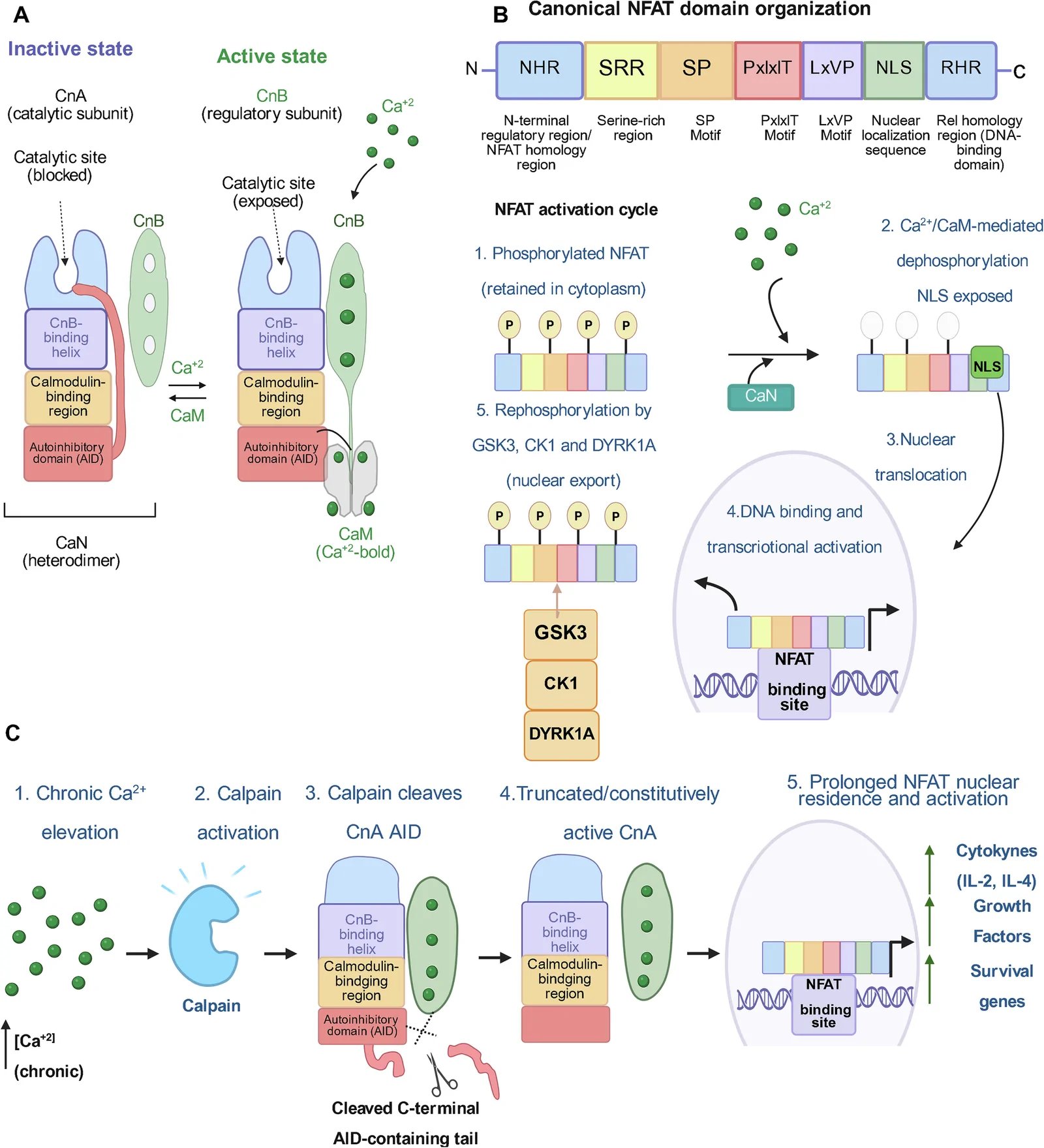
Structural organisation and regulation of the calcineurin/NFAT signalling axis. **A** Calcineurin (CaN) is a Ca²⁺/calmodulin-dependent serine/threonine phosphatase composed of a catalytic A subunit (CnA) and a regulatory B subunit (CnB). In the inactive state, the autoinhibitory domain (AID) of CnA blocks the catalytic site. Upon elevation of intracellular Ca²⁺, Ca²⁺ binding to CnB and calmodulin promotes calmodulin association with CnA, displacement of the AID, and exposure of the catalytic site, resulting in CaN activation. **B** Canonical NFAT proteins contain an N-terminal regulatory region/NFAT homology region (NHR), serine-rich regions and SP motifs, CaN-docking motifs (PxIxIT and LxVP), a nuclear localisation sequence (NLS), and a Rel homology region (RHR) responsible for DNA binding. In resting cells, phosphorylated NFAT is retained in the cytoplasm. Ca²⁺/CaN-dependent dephosphorylation exposes the NLS and promotes NFAT nuclear translocation, DNA binding, and transcriptional activation. Signal termination occurs through rephosphorylation by kinases including GSK3, CK1, and DYRK1A, which drives NFAT nuclear export. **C** Under pathological conditions, chronic Ca²⁺ elevation can activate calpain proteases. Calpain-mediated cleavage of the calcineurin AID may generate truncated, persistently active CaN species, thereby prolonging NFAT nuclear residence and reinforcing CaN/NFAT-dependent transcriptional programmes. This mechanism provides a disease-relevant route by which sustained Ca²⁺ stress may uncouple CaN activity from physiological temporal control. Created with Biorender.

Calcineurin (CaN), also known as protein phosphatase 2B (PP2B), is a Ca²⁺/calmodulin-dependent serine/threonine phosphatase originally identified in brain tissue as a major calmodulin-binding protein [16]. Structurally, CaN is a heterodimer composed of a catalytic A subunit (CnA) and a regulatory B subunit (CnB) (Fig. 1A). Three CnA isoforms (CnAα, CnAβ and CnAγ) have been described, of which CnAα and CnAβ are predominantly expressed in the brain, whereas CnAγ shows restricted expression in testis and peripheral tissues [17].

The CnA subunit contains the catalytic domain, a calmodulin-binding region and a C-terminal autoinhibitory domain (AID) (Fig. 1A). Under basal conditions, the AID occludes the active site, maintaining CaN in an inactive state. Elevation of intracellular Ca²⁺ leads to Ca²⁺ binding to CnB and calmodulin, promoting calmodulin association with CnA and displacement of the AID, thereby activating the phosphatase [18, 19] (Fig. 1A). This activation mechanism enables CaN to decode both the amplitude and duration of Ca²⁺ signals, distinguishing transient physiological Ca²⁺ fluctuations from sustained pathological elevations.

In the CNS, CaN is widely expressed in neurons, astrocytes and microglia, where it regulates processes including synaptic plasticity, neuronal excitability, and inflammatory responses [12, 20]. At synapses, CaN is a critical mediator of activity-dependent signalling, particularly in pathways associated with long-term depression (LTD), where modest and sustained Ca²⁺ increases preferentially activate phosphatase-driven responses over kinase-dominated pathways [21].

CaN substrates bind to discrete non-catalytic docking surfaces on the CnA subunit via conserved PxIxIT and LxVP motifs. These interactions promote substrate recruitment and proper positioning within the catalytic cleft, thereby conferring specificity and efficiency to Ca²⁺-dependent dephosphorylation [22, 23].

CaN activity is tightly controlled by multiple regulatory mechanisms that are especially refined in neural tissue. Members of the regulator of CaN (RCAN) family, including RCAN1/DSCR1, act as feedback inhibitors of CaN activity and thereby buffer excessive Ca²⁺-dependent signalling [24]. An additional layer of regulation is provided by proteins such as Cabin1, which interacts with CaN and restricts NFAT activation under specific cellular contexts [25]. CaN function is also sensitive to the cellular redox state, with RCAN1, the best-characterised member of the calcipressin family, providing short-term protection against acute oxidative stress and other Ca²⁺-mediated stresses [26]. A further mechanism by which Ca²⁺ stress may regulate CaN activity in neurons involves Ca²⁺-activated calpain proteases. Calpain-1 and calpain-2 are Ca²⁺-regulated cysteine proteases whose activation results in the removal of autoinhibitory domain leading to constitutive CaN activation (Fig. 1B) [27].

Together, these regulatory layers ensure that CaN activity remains closely coupled to physiological Ca²⁺ signalling while preventing chronic overactivation. Disruption of this balance, most commonly through sustained alteration of Ca²⁺ homoeostasis, can result in prolonged CaN activation and altered downstream signalling, creating conditions under which CaN-dependent pathways may contribute to synaptic dysfunction and neuroinflammatory responses observed in neurodegenerative diseases [8, 28].

## Nuclear factor of activated T cells: structure, regulation and functional roles in the CNS

The nuclear factor of activated T cells (NFAT) family comprises a group of transcription factors originally characterised in immune cells as key regulators of Ca²⁺-dependent gene expression. Subsequent studies demonstrated that NFAT proteins are also expressed in the CNS, where they mediate activity-dependent transcriptional programmes in neurons and glial cells [29].

In mammals, the NFAT family consists of five members: NFATc1 (NFAT2), NFATc2 (NFAT1), NFATc3 (NFAT4), NFATc4 (NFAT3), and NFAT5, also known as tonicity-responsive enhancer binding protein (TonEBP). The canonical NFAT proteins (NFATc1-c4) share a conserved regulatory architecture that confers sensitivity to Ca²⁺/CaN signalling, whereas NFAT5 lacks CaN-binding motifs and is regulated primarily by osmotic stress [30, 31].

Canonical NFAT proteins are characterised by two major domains: an N-terminal regulatory region, termed the NFAT homology region (NHR), and a C-terminal Rel homology region (RHR) that mediates DNA binding. The NHR contains multiple serine-rich regions and serine-proline motifs that are targets of kinases that are active in resting cells, notably GSK3, CK1, and DYRK1A, with additional regulation by PKA in response to cAMP signalling., as well as the two conserved CaN-docking motifs (PxIxIT and LxVP) and a nuclear localisation signal (NLS) [22, 23] (Fig. 1C).

Under resting conditions, NFAT proteins are highly phosphorylated and retained in the cytoplasm. Elevation of intracellular Ca²⁺ activates CaN, which dephosphorylates specific serine clusters within the NHR, resulting in exposure of the NLS and rapid nuclear translocation of NFAT [30, 32] (Fig. 1C). Termination of NFAT signalling occurs when Ca²⁺ levels decline, allowing rephosphorylation by GSK3β, CK1 and DYRK1A, thereby promoting nuclear export and restoring cytoplasmic sequestration [33]. This activation-deactivation cycle enables NFAT to function as a decoder of Ca²⁺ signal duration rather than amplitude, preferentially responding to sustained Ca²⁺ elevations.

NFAT proteins bind DNA with relatively low affinity as monomers and typically require cooperative interactions with other transcription factors to achieve stable promoter occupancy. The best-characterised partners are members of the AP-1 family (Fos/Jun), with which NFAT forms composite transcriptional complexes at regulatory elements of target genes [34]. This requirement for transcriptional cooperation provides an important layer of context specificity, integrating Ca²⁺ signals with parallel pathways activated by growth factors, stress responses or inflammatory cues.

Importantly, disruption of NFAT-AP-1 coupling can alter transcriptional outcomes, allowing NFAT to engage alternative gene programmes. This principle, well established in immune cells, is increasingly recognised as relevant in neural contexts, where NFAT-dependent transcription may diverge depending on cell type, developmental stage and signalling milieu [35].

Within the CNS, canonical NFAT isoforms (NFATc1-c4) are differentially expressed in neuronal and glial populations, although their distribution is partially overlapping and varies across developmental stages and experimental models.

NFATc3 and NFATc4 have been detected in neurons and have been implicated in activity-dependent transcriptional responses. Experimental studies have linked CaN/NFAT signalling in neurons to processes such as neurite extension, synaptic plasticity, and regulation of activity-responsive genes, including brain-derived neurotrophic factor (BDNF) and growth-associated protein 43 (GAP-43) [36]. However, most evidence derives from in vitro systems or defined experimental paradigms, and comprehensive isoform-specific mapping in vivo remains incomplete.

NFATc1 and NFATc2 are also expressed in the CNS and have been reported in astrocytes and microglia, where CaN/NFAT signalling has been associated with regulation of inflammatory mediators and glial activation markers [37, 38]. In microglia in particular, NFAT-dependent transcription has been involved in cytokine production in response to inflammatory stimuli [39]. Nevertheless, these associations do not imply strict cell-type specificity, as multiple NFAT isoforms can be detected in both neuronal and glial compartments [40–42].

Isoform-specific functions remain ill-defined, because many studies rely on pharmacological CaN inhibition or overexpression, which do not discriminate between NFAT-family members [43]. Moreover, functional redundancy among NFAT isoforms has been documented in other tissues and may also apply within the CNS. Thus, direct comparative analyses across cell types and disease models would be required to clarify these aspects of the NFAT biology [44].

Under physiological conditions, CaN/NFAT signalling contributes to Ca²⁺-dependent transcriptional programmes in both neurons and glial cells. In neurons, NFAT activation has been associated with activity-dependent gene regulation, whereas in glial cells it has been linked to inflammatory and reactive responses. In pathological contexts characterised by sustained Ca²⁺ dysregulation or chronic inflammatory signalling, increased NFAT nuclear localisation has been reported in both neuronal and glial populations [12, 13]. Although current evidence supports an association between NFAT activation and neurodegenerative conditions, NFAT do not appear to function as initiating drivers, and their contribution depends on cell type, stimulus, disease model and the isoform-specific.

## Physiological functions of the Ca^2+^/NFAT pathway in the CNS

### Synaptic plasticity

Synaptic plasticity encompasses activity-dependent modifications of synaptic strength that underlie learning, memory and circuit refinement. Long-term potentiation (LTP) and long-term depression (LTD) represent two well-characterised forms of plasticity, distinguished by the magnitude and temporal profile of intracellular Ca²⁺ signals triggered by synaptic activity [21].

In canonical hippocampal models, high-frequency stimulation generates rapid, high-amplitude postsynaptic Ca²⁺ transients through NMDA receptors, preferentially activating kinase-dominated pathways such as Ca^2+^/Calmodulin-dependent protein kinase II (CaMKII) and the MAPK/ERK cascade [21, 45]. These events lead to AMPA receptor phosphorylation and insertion into the post-synaptic membrane of dendritic spines and engage LTP transcriptional programmes that support the maintenance of late-phase LTP [46, 47]. In contrast, lower-frequency but more sustained Ca²⁺ increases preferentially activate phosphatase-driven signalling pathways, particularly CaN. Through a CaN-PP1 cascade, AMPA receptors are dephosphorylated, promoting their internalisation from the postsynaptic membrane and leading to expression of NMDAR-dependent LTD [20, 21].

Therefore, CaN is considered a critical mediator of NMDAR-dependent LTD induction. Its role in synaptic weakening is supported by extensive electrophysiological and pharmacological evidence demonstrating that CaN inhibition disrupts LTD in hippocampal CA1 neurons while leaving LTP largely intact [48, 49].

The involvement of NFAT downstream of CaN in synaptic plasticity is less well established than that of CaMKII or CaN itself. NFAT activation depends on sustained Ca²⁺ elevations and CaN activity, and NFAT-dependent transcription has been implicated in the regulation of genes associated with structural remodelling and neuronal excitability, including RCAN1 and, in some contexts, BDNF [50, 51]. These observations support a role for NFAT in consolidating longer-term adaptations associated with synaptic activity rather than in the rapid induction of synaptic weight changes.

Importantly, most forms of early-phase LTP and LTD occur independently of transcription and rely on post-translational mechanisms. NFAT-dependent transcription is therefore unlikely to be required for the immediate expression of synaptic plasticity. Instead, available evidence suggests that NFAT may contribute to activity-dependent transcriptional programmes engaged during late-phase plasticity or longer-term adaptations to sustained neuronal activity, although direct causal links to synaptic consolidation remain incompletely defined [52–54].

Experimental evidence linking NFAT directly to synaptic plasticity is largely derived from genetic manipulation or chronic pharmacological modulation of CaN/NFAT signalling. In these contexts, altered NFAT activity has been associated with changes in dendritic spine density, activity-dependent synaptic pruning, and structural aspects of synaptic maintenance [51]. However, pharmacological inhibition of CaN affect multiple downstream pathways beyond NFAT, and even genetic approaches may engage compensatory mechanisms [55]. These factors complicate the attribution of observed phenotypes specifically to NFAT-dependent transcription rather than to broader CaN-mediated effects [56, 57].

**Fig. 2:**
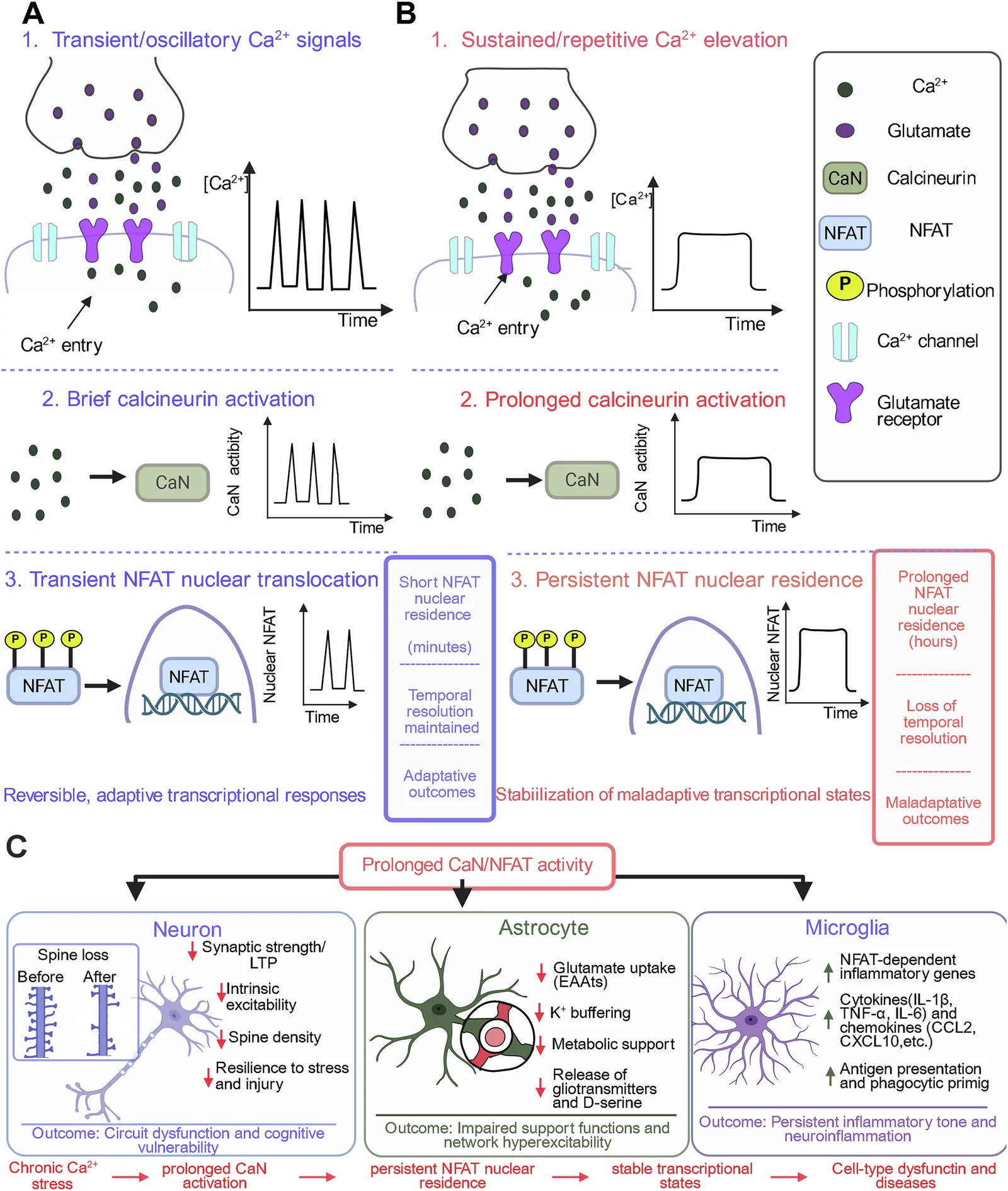
Ca^2+^/CaN/NFAT signalling in health and disease. **A** Physiological Ca^2+^ signalling is characterised by transient oscillations that drive brief CaN activation, transient NFAT nuclear translocation, and reversible transcriptional programmes that support adaptation. **B** Pathological Ca^2+^ stress produces sustained CaN activation and prolonged NFAT nuclear residence, leading to stable, maladaptive transcriptional states. **C** Persistent CaN/NFAT activity induces distinct, cell-type-specific programmes that collectively promote dysfunction across the CNS. Created with Biorender.

These limitations are particularly relevant when extrapolating physiological roles to pathological contexts. In neurodegenerative conditions, chronic Ca²⁺ dyshomeostasis can shift CaN activity from transient, activity-coupled signalling toward sustained activation. Under such conditions, prolonged NFAT activity has been linked to transcriptional changes correlated with synaptic weakening, loss of dendritic spines and altered excitability. Although supported by correlative and mechanistic studies, these findings do not establish NFAT activation as an initiating driver of pathology. Rather, maladaptive outcomes are likely to reflect disruption of the temporal constraints that normally restrict CaN/NFAT signalling during physiological synaptic activity [11, 13] (Fig. 2A, B).

Taken together, current evidence supports a model in which CaN plays a direct and well-established role in LTD and synaptic weakening, whereas NFAT functions as a downstream transcriptional integrator that may contribute to longer-term synaptic and structural adaptations [56]. The extent to which NFAT-dependent transcription is engaged during physiological synaptic plasticity likely depends on the duration, spatial distribution and cellular context of Ca²⁺ signals, emphasising the importance of temporal regulation in distinguishing adaptive from maladaptive signalling outcomes [58].

### Neurodevelopment and adult neurogenesis

The Ca²⁺/CaN/NFAT signalling pathway plays well-established roles during embryonic development, where it regulates cell-fate specification, tissue morphogenesis and differentiation across multiple organ systems, including the cardiovascular, immune and nervous systems [59, 60]. In the developing CNS, genetic and cellular models have implicated CaN/NFAT signalling in neuronal differentiation, axonal and dendritic growth, and activity-dependent refinement of neural circuits [61].

During neurodevelopment, Ca²⁺ signals arising from growth factor receptors, voltage-gated Ca²⁺ channels and neurotransmitter receptors activate CaN thereby regulating NFAT nuclear translocation and transcriptional activity [57]. Genetic and conditional knockout studies demonstrate that NFAT family members contribute to neural development in isoform- and context-specific manners [60, 62]. However, the functional consequences of NFAT activation are highly dependent on developmental stage and cellular context. In some experimental systems, NFAT activity promotes neuronal differentiation and neurite extension, whereas in others it has been implicated in influencing progenitor cell fate decisions, including glial lineage specification. These findings underscore the importance of developmental stage, cellular identity and signalling environment in shaping NFAT-dependent functional outcomes [63].

In the adult brain, the CaN/NFAT pathway is expressed in restricted neurogenic niches, most prominently in the subgranular zone of the hippocampal dentate gyrus. Adult hippocampal neurogenesis relies on tightly regulated Ca²⁺-dependent signalling to control neural stem and progenitor cell proliferation, survival and differentiation [51, 64]. Experimental evidence indicates that NFAT-dependent transcription can be engaged downstream of neurotrophin in neural progenitor populations, suggesting a potential role for this pathway in aspects of adult neurogenesis [61].

However, the contribution of NFAT to adult neurogenesis should be interpreted cautiously. Changes in NFAT activity correlate with altered proliferation or differentiation of neural progenitors in experimental models, often under conditions of sustained signalling manipulation or genetic perturbation. Direct evidence demonstrating that NFAT acts as a physiological rate-limiting determinant of adult neurogenesis under basal conditions remains limited, and functional redundancy among transcriptional regulators may constrain the impact of NFAT signalling in vivo [61, 65, 66].

Ageing and neurodegenerative conditions are associated with impaired adult hippocampal neurogenesis [67]. Altered Ca²⁺ homoeostasis and increased CaN activity have been proposed as contributing factors in this decline [13]. In this context, NFAT activation has been implicated in transcriptional changes accompanying reduced neurogenic capacity. However, available data do not support a simple reactivation of embryonic developmental programmes in the adult diseased brain. Sustained Ca²⁺/CaN/NFAT signalling may influence the balance between proliferation, differentiation and survival within adult neural stem cell niches, particularly when temporal regulation of signalling is disrupted [68].

In conclusion, CaN/NFAT signalling seems to contribute to neurodevelopmental and adult neurogenic processes in a context-dependent manner. NFAT functions as a Ca²⁺-responsive transcriptional regulator capable of shaping neural differentiation and plasticity when appropriately constrained. In adult neurogenesis and disease-related states, however, its role appears modulatory rather than deterministic. This distinction is critical for interpreting NFAT activation in pathological settings and for evaluating the therapeutic implications of targeting this pathway.

## Ca^2+^/NFAT signalling and neuroinflammation

Neuroinflammation is a prominent feature of many neurodegenerative diseases and reflects the activation of resident glial cells, primarily microglia and astrocytes. Although these cells do not generate action potentials, they rely extensively on intracellular Ca²⁺ signalling to regulate gene expression, metabolic adaptation and inflammatory responses [40, 41]. Dysregulation of Ca²⁺ homoeostasis in glial cells has been proposed as an important contributing mechanism to sustained neuroinflammatory states [12].

Astrocytes respond to diverse stimuli, including neurotransmitters, extracellular ATP and inflammatory mediators, through Ca²⁺ elevations that can engage Ca²⁺-dependent transcriptional pathways. Experimental studies demonstrate that mechanical injury, purinergic signalling and glutamatergic dysregulation can activate the CaN/NFAT pathway in astrocytes [69, 70]. NFAT activation in astrocytes has been associated with changes in gene expression that influence neuronal excitability and synaptic function [36] (Fig. 2C).

It functions as a modulatory pathway that integrates Ca²⁺ signals generated downstream of neuronal activity or tissue damage. Importantly, astrocytic inflammatory gene expression is strongly regulated by NF-κB and STAT signalling pathways, and current evidence does not support NFAT as a primary or independent driver of astrocyte-mediated neuroinflammation [12, 69].

Microglia serve as the innate immune cells of the central nervous system and exhibit highly dynamic activation states in response to tissue perturbation. Canonical microglial activation is initiated through pattern recognition receptors, such as Toll-like receptors (TLRs), which engage NF-κB and MAPK signalling cascades to induce pro-inflammatory gene expression [37, 71].

In parallel, sustained increases in intracellular Ca²⁺ can activate CaN and promote NFAT nuclear translocation in microglia. Prolonged Ca²⁺ signalling in immune cells, leading to NFAT activation, is often maintained through store-operated Ca²⁺ entry (SOCE), mediated by STIM and ORAI1 proteins, a mechanism well characterised in peripheral leucocytes and increasingly reported in microglial systems [72, 73]. NFAT activation in microglia has been shown to contribute to the expression of selected cytokines and chemokines, including TNF-α, IL-1β and MCP-1, particularly under conditions of sustained inflammatory stimulation [39] (Fig. 2C).

As in astrocytes, NFAT signalling in microglia does not operate in isolation. NF-κB remains the principal transcriptional driver of acute inflammatory responses, whereas NFAT appears to modulate the magnitude, persistence and cellular consequences of inflammation. Consistent with this division of labour, pharmacological or genetic inhibition of NFAT attenuates, but does not abolish, microglial inflammatory responses [38, 74].

Several Ca²⁺-permeable receptors expressed by microglia may reinforce NFAT activation during neuroinflammation. Among these, the purinergic P2X7 receptor (P2X7R), activated by high extracellular ATP concentrations released during tissue damage, induces robust Ca²⁺ influx that can support CaN signalling promoting transcriptional programmes associated with inflammatory amplification [75]. While NFAT has been implicated in the transcriptional regulation of inflammatory mediators downstream of P2X7R activation, the extent to which NFAT directly regulates P2X7R expression or establishes feed-forward loops that enhances microglial responsiveness under chronic inflammatory conditions remains to be fully defined [76].

Beyond cytokine production, NFAT signalling has also been associated with transcriptional regulation of genes linked to the cell cycle, including c-MYC, in immune cells, and similar mechanisms have been proposed in microglia [42], where this proliferative response may contribute to the expansion of reactive populations in chronic neurodegeneration [77].

In excitotoxic neuronal models, calpain-mediated cleavage of the CnA autoinhibitory domain generates a truncated CaN species with prolonged activity, thereby converting elevated Ca²⁺ exposure into sustained CaN signalling and neuronal injury [78]. This proteolytic mechanism provides an additional link between chronic Ca²⁺ dysregulation and persistent CaN/NFAT activation in neurodegenerative contexts (Fig. 2C).

Taken together, Ca²⁺/NFAT signalling may act as a modulatory component of neuroinflammatory responses rather than as a primary initiating pathway. NFAT integrates sustained Ca²⁺ signals with innate immune signalling to influence the persistence and qualitative features of glial activation [37]. Disruption of the temporal constraints that normally restrict Ca²⁺/CaN/NFAT signalling in glia and neurons has been proposed as a mechanism contributing to maladaptive neuroinflammation in neurodegenerative disease [38, 79].

## CaN/NFAT pathway dysfunction in neurodegenerative diseases

### Alzheimer’s disease

Alzheimer’s Disease (AD) is characterised by progressive cognitive decline associated with synaptic dysfunction, neuronal loss, extracellular accumulation of amyloid-β (Aβ) peptides, and intracellular aggregation of hyperphosphorylated tau [4]. In addition to these hallmark pathologies, accumulating evidence indicates that disruption of intracellular Ca²⁺ homoeostasis is a consistent feature of AD and contributes to both neuronal dysfunction and glial activation [7].

**Fig. 3:**
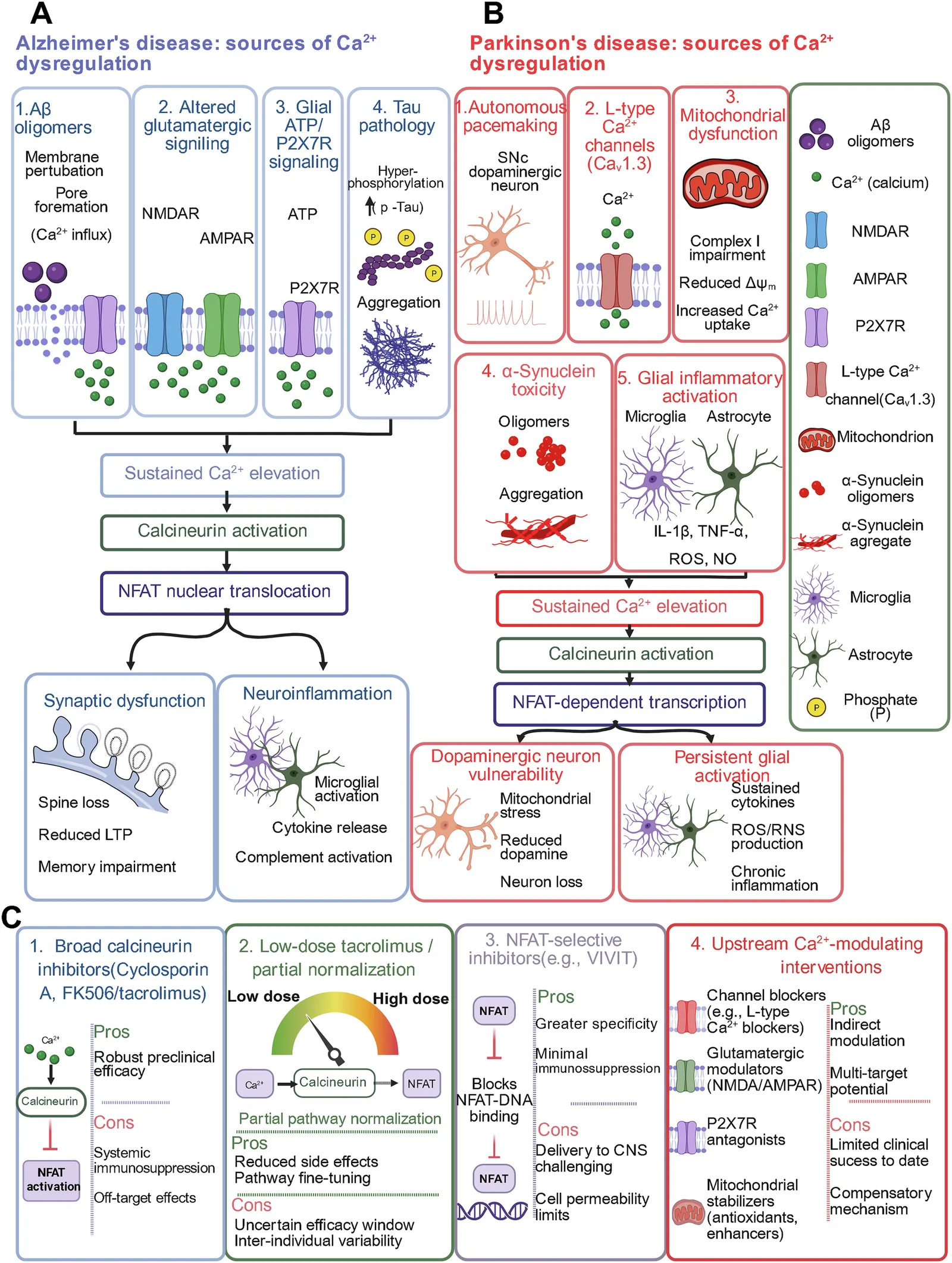
Disease-specific routes converging on Ca^2+^/CaN/NFAT signalling in neurodegeneration. **A** Alzheimer’s disease: distinct sources of Ca^2+^ dysregulation drive sustained Ca^2+^ elevation, CaN activation, and NFAT translocation, leading to synaptic dysfunction and neuroinflammation. **B** Parkinson’s disease: multiple mechanisms create a chronic Ca^2+^ burden that activates CaN and NFAT-dependent transcription, promoting dopaminergic neuron vulnerability and persistent glial activation. **C** Therapeutic opportunities and limitations for targeting the Ca^2+^/CaN/NFAT axis across neurodegenerative diseases. Created with Biorender.

Aβ oligomers perturb neuronal Ca²⁺ handling through multiple mechanisms, including altered NMDA receptor activity, changes in AMPA receptor composition, and impaired intracellular Ca²⁺ buffering. These alterations can result in sustained elevations of intracellular Ca²⁺, particularly in vulnerable neuronal populations. Under these conditions, increased or sustained CaN activation has been reported [80] (Fig. 3A).

In addition to receptor-mediated mechanisms, Aβ assemblies can directly affect membrane permeability. Early work showed that β-amyloid peptides destabilise neuronal Ca²⁺ homoeostasis and increase vulnerability of human cortical neurons to excitotoxic stimuli [81]. Subsequent biophysical and electrophysiological studies supported the idea that Aβ1-42 can form irregular cation-permeable channel-like structures in lipid bilayers, with conductance properties influenced by solvent conditions and pH [82]. Consistent with this view, Aβ-induced membrane perturbation in hippocampal neurons has been proposed to create leaky bilayer states and abnormal Ca²⁺ influx, providing an additional route by which extracellular Aβ burden can be translated into chronic intracellular Ca²⁺ stress [83].

Experimental studies in AD models consistently show increased CaN activity in affected brain regions, and pharmacological or genetic reduction of CaN activity attenuates synaptic deficits and neuronal vulnerability [13, 84]. These findings support a direct role for CaN as a mediator linking Ca²⁺ dysregulation to synaptic and cellular dysfunction in AD. Consistent with prolonged Ca²⁺/CaN activation, increased nuclear localisation of NFAT has been observed in both neurons and glial cells in AD models [11, 80].

In neurons, NFAT-dependent transcription has been associated with changes in genes involved in synaptic stability, dendritic structure and excitability [85–87]. However, direct evidence that NFAT activation alone is sufficient to drive neuronal death or synaptic loss in AD remains limited. Most studies rely on CaN inhibition or broad NFAT suppression, approaches that affect multiple CaN-dependent pathways simultaneously [11].

The amyloidogenic processing of amyloid precursor protein (APP) by β-site APP-cleaving enzyme 1 (BACE1) is a central pathogenic mechanism in AD. NF-κB signalling has been clearly implicated in transcriptional regulation of BACE1 [88]. In contrast, the involvement of NFAT in BACE1 regulation is indirect and context-dependent. Although modulation of NFAT activity can influence BACE1 expression in cellular and animal models, direct binding of NFAT to the BACE1 promoter has not been conclusively demonstrated [89]. Collectively, these observations suggest that Ca²⁺/CaN/NFAT signalling modulates amyloidogenic pathways under pathological conditions, rather than directly initiating Aβ production. Such modulation may contribute to feed-forward interactions between Ca²⁺ dysregulation, synaptic dysfunction and amyloid pathology without implying linear causality.

Neuroinflammation is a prominent feature of AD and is mediated by sustained activation of microglia and astrocytes. In glial cells, NFAT activation occurs downstream of Ca²⁺ signals generated by inflammatory stimuli, extracellular ATP and purinergic receptor activation [12, 37].

In microglia, NFAT contributes to the expression of selected cytokines and chemokines, including TNF-α and IL-1β, particularly under conditions of chronic stimulation. However, NF-κB remains the primary driver of acute inflammatory gene expression, whereas NFAT is thought to modulate the persistence and magnitude of inflammatory responses [38, 90]. In astrocytes, NFAT activation has been linked to transcriptional programmes that influence neuronal excitability and synaptic function, potentially contributing to pathological dysfunction in AD [80].

Tau hyperphosphorylation and aggregation represent a second major pathological axis in AD. Although NFAT does not directly regulate tau expression or phosphorylation, CaN can influence phosphorylation dynamics through multiple mechanisms, including direct dephosphorylation of tau and modulation of kinase activity such as that of GSK3β [91]. In AD mouse models, CaN inhibition reduces both Aβ burden and tau pathology, even though these effects cannot be attributed unequivocally to NFAT inhibition and likely reflect broader modulation of CaN-dependent pathways [84]. Collectively, available evidence suggests that Ca²⁺ dyshomeostasis and sustained CaN activation represent important contributors to synaptic dysfunction and neuroinflammation in AD. NFAT activation emerges as a downstream transcriptional response that integrates chronic Ca²⁺ signals in neurons and glial cells. Under pathological conditions, prolonged engagement of this pathway may reinforce maladaptive gene expression programmes, suggesting a context-dependent modulatory role for NFAT and highlighting the relevance of temporal and spatial control of Ca²⁺/CaN signalling.

### Parkinson’s disease

Parkinson’s Disease (PD) is a progressive neurodegenerative disorder characterised by the selective loss of dopaminergic neurons in the substantia nigra pars compacta and by the accumulation of misfolded α-synuclein in Lewy bodies and Lewy neurites [92–94]. In addition to protein aggregation, PD pathology involves mitochondrial dysfunction, oxidative stress and a prominent neuroinflammatory component, all of which contribute to neuronal vulnerability [6, 92].

A defining physiological feature of nigral dopaminergic neurons is their autonomous pacemaker activity, which relies in part on L-type voltage-gated Ca²⁺ channels, particularly Cav1.3. This intrinsic activity results in sustained Ca²⁺ influx and imposes a high metabolic burden, increasing mitochondrial stress and reactive oxygen species (ROS) production [95–97]. In PD, mitochondrial impairment and α-synuclein-mediated toxicity further compromise Ca²⁺ handling, predisposing neurons to elevated cytosolic Ca²⁺ stress (Fig. 3B).

In this context, increased or sustained CaN activation has been reported in vulnerable neurons. Experimental models of PD demonstrate increased CaN activity, and pharmacological inhibition of CaN attenuates dopaminergic neuron loss and motor deficits in toxin-based PD models [98].

NFAT nuclear translocation has been reported in both neurons and glial cells exposed to α-synuclein aggregates or inflammatory stimuli [99, 100]. In neurons, NFAT-dependent transcription has been associated with alterations in genes regulating synaptic function and neuronal resilience. However, direct evidence that neuronal NFAT activation alone is sufficient to induce dopaminergic neuron death is limited. As in AD, most experimental evidence relies on CaN inhibition or broad NFAT suppression, which affects multiple CaN substrates and complicates attribution of neuronal phenotypes specifically to NFAT.

Neuroinflammation is a key contributor to PD progression. Misfolded or aggregated α-synuclein can activate microglia through TLR2 and TLR4, leading to NF-κB-driven transcription of pro-inflammatory cytokines [101, 102]. In parallel, inflammatory signalling and extracellular ATP release promote sustained Ca²⁺ elevations in microglia, providing conditions permissive for CaN/NFAT activation.

NFAT signalling in microglia has been associated with the expression of selected inflammatory mediators and with proliferative responses under chronic inflammatory conditions [42, 74]. However, NF-κB remains the dominant driver of acute inflammatory gene expression, whereas NFAT appears to modulate the persistence and amplification of inflammatory responses. Experimental attenuation of NFAT signalling reduces, but does not abolish, neuroinflammatory activation in PD models, supporting a modulatory role [74]. Sustained neuroinflammation can exacerbate α-synuclein pathology by promoting oxidative stress and impairing proteostatic mechanisms, thereby reinforcing neuronal injury. Conversely, increased α-synuclein burden further activates glial cells, establishing a feed-forward loop that may accelerate disease progression [103, 104]. Ca²⁺/CaN/NFAT signalling likely participates in this loop by integrating prolonged Ca²⁺ signals into transcriptional programmes associated with chronic glial activation.

Together the data suggest that altered Ca²⁺ homoeostasis may represent a central determinant of neuronal vulnerability in PD, driven by intrinsic pacemaker activity, mitochondrial dysfunction and α-synuclein toxicity. CaN activation constitutes a key molecular consequence of sustained Ca²⁺ stress, while NFAT functions as a downstream transcriptional regulator in both neurons and glial cells, contributing to maladaptive inflammatory and stress-response programmes under pathological conditions. Its relevance is therefore best understood as context-dependent and contingent upon chronic disruption of Ca²⁺ signalling control.

## Targeting Ca^2+^/CaN/NFAT signalling in neurodegeneration: opportunities and limitations

The Ca²⁺/CaN/NFAT axis represents an attractive therapeutic target because it integrates intracellular Ca²⁺ dynamics with transcriptional programmes implicated in synaptic dysfunction and neuroinflammation [74, 98, 105]. However, its central physiological roles in multiple cell types impose substantial constraints on therapeutic modulation, particularly in the CNS (Fig. 3C).

Classical CaN inhibitors (CNIs), such as cyclosporin A (CsA) and tacrolimus (FK506), effectively suppress CaN activity and NFAT-dependent transcription (Table 1). In experimental models of neurodegeneration, CNIs attenuate synaptic deficits, reduce neuroinflammatory markers and improve behavioural outcomes [13, 98], establishing CaN inhibition as a valid proof-of-principle strategy.

**Table 1:** Strategies to modulate the calcineurin-NFAT pathway.

Strategy	Representative agents	Molecular target	Key advantages	Current limitations and challenges	References
Classical CNIs	Cyclosporine A (CsA), Tacrolimus (FK506)	Calcineurin (CaN) Catalytic Site	Well-establishe clinical use; potent immunosuppression	High toxicity (nephro/neuro); blocks all CaN functions (off-target)	(Cohen et al. [116])
Selective Peptide	VIVIT, 11R-VIVIT	CaN-NFAT Docking Sites (PxIxIT/LxVP)	High selectivity; preserves non-NFAT CaN functions	Poor Blood-Brain Barrier (BBB) permeability; requires delivery vectors	(Gal et al. [19])
Upstream Ca²⁺ Modulation	L-type CCBs (Isradipine), NMDAR antagonists	Intracellular Ca²⁺ influx	Indirectly reduces pathway hyperactivation; existing clinical safety data	May lack specificity for the NFAT pathway; systemic cardiovascular effects	(Little [108])
Endogenous Modulators	CABIN-derived peptides	Physiological CaN/NFAT interaction	Mimics natural regulatory mechanisms; potentially lower toxicity	Complex dose-response relationship; difficult to deliver exogenously	(Lee et al. [117])
Genetic Approaches	siRNA, Viral vectors (AAV)	NFAT mRNA/Isoform expression	High specificity for isoforms (e.g., NFATc2); long-term modulation	Delivery challenges; potential for permanent genomic/transcriptional changes	(Si et al. [114])
Emerging Delivery	Nanoparticles, Exosomes	BBB transport systems	Enhanced CNS penetration; targeted delivery to glia or neurons	Still in early preclinical stages; high production complexity and cost	(Jin et al. [115])

This table summarises therapeutic approaches targeting the CaN-NFAT signalling axis, detailing representative agents, molecular targets, principal advantages, and major limitations. The strategies include classical calcineurin inhibitors (CNIs), selective peptide-based modulators, Ca²⁺ modulating interventions, endogenous regulatory mechanisms, and genetic approaches. Emerging delivery platforms, such as nanoparticles and exosomes, are noted for their ability to improve central nervous system (CNS) penetration and enable cell-type-specific targeting. However, these technologies are in early preclinical development and meet considerable manufacturing and cost challenges. The table offers a comparative overview of continuing efforts to develop safer and more effective modulators of CaN-NFAT activity in CNS-related disorders. References supporting each strategy are provided.

Nevertheless, CNIs lack pathway specificity and broadly interfere with CaN-dependent processes beyond NFAT signalling, including regulation of synaptic vesicle cycling, cytoskeletal dynamics and mitochondrial function [20]. Moreover, chronic CNI administration is associated with well-documented adverse systemic effects such as nephrotoxicity, hypertension and increased infection risk, rendering long-term use in chronic neurodegenerative diseases unlikely to be feasible in their current form [106].

Recent preclinical studies have begun to refine this view by testing whether low-dose or subclinical CNI regimens can normalise excessive CaN activity without producing full systemic immunosuppression. In AD model mice, long-term low-dose FK506 normalised CNS CaN activity, reduced neuroinflammatory and AD-associated phenotypes, and did so without blocking peripheral T-cell responses [83]. Similarly, in an aging canine model of AD, tacrolimus and related CaN/NFAT inhibitors preserved cognitive performance and brain health measures, supporting the concept that partial pathway normalisation may have preventive or disease-modifying potential [107]. These approaches partially attenuate peripheral toxicity and may improve long-term safety.

To overcome the non-specificity of CaN inhibition, efforts have focused on selectively disrupting CaN-NFAT interactions. Peptide inhibitors derived from NFAT docking motifs, such as VIVIT, selectively block NFAT dephosphorylation without globally suppressing CaN activity [22] (Table 1). In cellular and animal models, NFAT-selective inhibition reduces inflammatory gene expression and attenuates pathological phenotypes while preserving other CaN-dependent functions [74, 105, 107]. Despite these advantages, NFAT-selective strategies face significant translational barriers. Peptide inhibitors exhibit poor pharmacokinetic properties, limited blood-brain barrier penetration, suboptimal pharmacokinetics, and potential immunogenicity. Furthermore, NFAT family members display partial functional redundancy and context-dependent roles, raising concerns that incomplete or isoform-biased inhibition may yield variable outcomes. At present, NFAT-selective approaches remain primarily experimental tools rather than clinically actionable therapies [19].

An alternative strategy involves indirect modulation of CaN/NFAT signalling by targeting upstream Ca²⁺ sources (Table 1). In PD, blockade of L-type Ca²⁺ channels using dihydropyridines has been proposed to reduce Ca²⁺ burden in dopaminergic neurons and thereby limit CaN activation [108, 109]. Similarly, modulation of NMDA receptor activity and intracellular Ca²⁺ buffering has been explored in AD models [110, 111]. Although these approaches may attenuate pathological Ca²⁺ signalling, clinical trials targeting Ca²⁺ channels or glutamatergic receptors have yielded limited or inconclusive results, underscoring the difficulty of selectively dampening pathological Ca²⁺ elevations without disrupting physiological signalling essential for neuronal function [112, 113]. These outcomes highlight the limited predictive value of Ca²⁺-centric interventions when applied without precise temporal and cellular specificity.

A recurring challenge in targeting the Ca²⁺/CaN/NFAT pathway is its divergent role across neurons, astrocytes and microglia. While NFAT activation in glial cells may contribute to sustained neuroinflammation, NFAT-dependent transcription in neurons can support adaptive responses under physiological conditions. Global pathway inhibition therefore risks suppressing protective as well as maladaptive signalling.

Emerging strategies aimed at cell-type-specific modulation, such as viral vectors, nanoparticle-based delivery systems and gene regulatory approaches, offer conceptual solutions but remain technically immature for widespread therapeutic application in neurodegenerative disease [114, 115].

In summary, modulation of the Ca²⁺/CaN/NFAT pathway holds clear mechanistic relevance for neurodegenerative disease but presents substantial translational challenges. Broad CaN inhibition demonstrates efficacy in experimental models but is incompatible with long-term clinical use. NFAT-selective strategies provide improved specificity yet remain constrained by delivery and redundancy issues. Targeting upstream Ca²⁺ dysregulation may indirectly modulate CaN/NFAT signalling, but thus far has shown limited clinical success.

## Conclusions

The Ca²⁺/CaN/NFAT signalling axis functions in the CNS as a mechanism that translates the temporal structure of intracellular Ca²⁺ signals into sustained transcriptional responses. Although many Ca²⁺-dependent processes regulate rapid synaptic events, CaN/NFAT signalling is preferentially engaged during sustained or repetitive Ca²⁺ elevations, thereby linking prolonged cellular activity to long-term gene expression programmes.

In neurodegenerative disorders, persistent Ca²⁺ dysregulation reshapes this signalling module. Chronic activation of CaN alters the nuclear residence and transcriptional activity of NFAT in both neurons and glial cells. Under these conditions, NFAT may contribute to the stabilisation of transcriptional programmes that influence synaptic structure, inflammatory tone and cellular resilience. Its pathological relevance therefore appears to reside not in initiating Ca²⁺ stress, but in consolidating prolonged disturbances in Ca²⁺ homoeostasis into durable molecular states.

Across AD and PD, converging evidence suggests that CaN/NFAT signalling participates in the shift from adaptive stress responses to maladaptive persistence. In neurons, prolonged activation is associated with altered synaptic maintenance and increased vulnerability. In glial populations, NFAT contributes to the persistence and qualitative shaping of inflammatory responses. Although these effects are highly context-dependent, they reflect the shared principle that NFAT integrates the duration and intensity of Ca²⁺ signals into transcriptional outputs with extended temporal impact.

Neurodegeneration may therefore be interpreted not solely as a disorder of protein aggregation or inflammatory activation, but as a progressive failure in the temporal regulation of Ca²⁺-dependent signalling. Within this framework, CaN/NFAT does not function as a classical upstream trigger of pathology. Rather, it occupies a strategic position at the interface between transient cellular stress and long-term transcriptional stabilisation. When physiological termination mechanisms become progressively impaired, NFAT-dependent transcription may convert episodic Ca²⁺ disturbances into persistent molecular states that reinforce synaptic fragility and inflammatory tone. This perspective shifts the emphasis from pathway activation per se to the loss of signal resolution. The critical pathological event may not be excessive NFAT activity alone, but the inability of neural cells to disengage Ca²⁺-dependent transcription once stress becomes chronic. In this sense, CaN/NFAT signalling may act less as an initiator of degeneration and more as a molecular memory system that consolidates unresolved cellular stress into durable gene expression programmes.

Therapeutically, this implies that effective modulation of the Ca²⁺/CaN/NFAT axis will require temporal and cellular precision. Broad inhibition of CaN activity risk disrupting essential physiological signalling, whereas strategies aiming at restoring appropriate signal duration, threshold sensitivity or cell-type-specific engagement may hold great promise. Future research should therefore focus not only on pathway inhibition, but on defining how Ca²⁺ signal dynamics are encoded and stabilised by NFAT under physiological and pathological conditions.

We advocate that the field should move beyond asking whether CaN/NFAT is activated in neurodegeneration and instead define how, where and for how long the pathway remains engaged. Particularly important will be studies that separate receptor-mediated Ca²⁺ entry from membrane-disruptive Aβ actions, distinguish reversible CaN activation from calpain-dependent proteolytic activation, and test whether low-intensity pharmacological normalisation can preserve protective CaN functions while preventing maladaptive transcriptional persistence.

Understanding how temporal gating of Ca²⁺ signals becomes destabilised in ageing and disease may therefore be more informative than further cataloguing downstream transcriptional targets. Such an approach reframes therapeutic intervention, rather than suppressing the pathway globally, restoring physiological signal termination and cell-type-specific control may represent a more viable strategy for preventing the transition from adaptive stress responses to irreversible neurodegenerative states.
